# Minnesota’s Loss, Iowa’s Gain

**Published:** 1900-08

**Authors:** 


### MINNESOTA'S LOSS, IOWA'S GAIN.

THE calling of Dr. M. H. Reynolds, of Minnesota, to the deanship of the veterinary department at Ames, Iowa, is a serious loss to Minnesota, a great gain to Iowa.

In no State of our Union has there been more valuable work done in the past five years than has been accomplished in Minnesota under the direction of Dr. Reynolds. His strong position on the State Board of Health and the dissemination of knowledge along veterinary sanitary lines have brought excellent results and awakened a lasting interest in preventive medicine. The losses that have been prevented and the added knowledge gained in control work, the aroused interest all over the State, the excellent work done at the college station, in the agricultural college, through bulletins, the press, and every other available channel are of so wide-spreading interest, of such vast importance, that we cannot believe the people of Minnesota will relinquish his connection with their State work without a strong effort and plea to retain him.

Iowa's gain will be a great one, in that he brings to the college work a cultured mind, one strong in discipline, tireless in work, broad and conservative in all his teachings and doings, and adding to the college a strength that will enlarge its sphere of usefulness, enrich its students, and save through many channels great losses to the State.

We are more than sorry to see Dr. Reynolds leave Minnesota at a time when the fruits of his well-laid plans are about to be garnered, and when his directing skill is of the greatest import.

We congratulate the State College of Iowa on obtaining so valuable an addition to her teaching-staff; the State in general in having within her borders another well-trained expert in

State veterinary control work, and the college so capable and strong a teacher.

